# Automated feature-based grading and progression analysis of diabetic retinopathy

**DOI:** 10.1038/s41433-021-01415-2

**Published:** 2021-03-17

**Authors:** Lutfiah Al-Turk, James Wawrzynski, Su Wang, Paul Krause, George M. Saleh, Hend Alsawadi, Abdulrahman Zaid Alshamrani, Tunde Peto, Andrew Bastawrous, Jingren Li, Hongying Lilian Tang

**Affiliations:** 1 Department of Statistics, Faculty of Sciences, King Abdulaziz University, Jeddah, Saudi Arabia; 2 NIHR Biomedical Research Centre at Moorfields Eye Hospital and the UCL Institute of Ophthalmology, London, United Kingdom; 3 Department of Computer Science, University of Surrey, Guildford, Surrey UK; 4 Faculty of Medicine, King Abdulaziz University, Jeddah, Saudi Arabia; 5 Ophthalmology Department, Faculty of Medicine, University of Jeddah, Jeddah, Saudi Arabia; 6 Medical Retina in Belfast Health and Social Care Trust; Northern Irish Diabetic Eye Screening Programme, Queen’s University Belfast, Belfast, NI UK; 7 International Centre for Eye Health, Department of Clinical Research, Faculty of Infectious and Tropical Diseases, London School of Hygiene and Tropical Medicine London, London, UK; 8 7th Medical Center of PLA General Hospital, Diabetes Professional Committee of China, Geriatric Health Association, Beijing, PR China

**Keywords:** Diagnosis, Medical imaging

## Abstract

**Background:**

In diabetic retinopathy (DR) screening programmes feature-based grading guidelines are used by human graders. However, recent deep learning approaches have focused on end to end learning, based on labelled data at the whole image level. Most predictions from such software offer a direct grading output without information about the retinal features responsible for the grade. In this work, we demonstrate a feature based retinal image analysis system, which aims to support flexible grading and monitor progression.

**Methods:**

The system was evaluated against images that had been graded according to two different grading systems; The International Clinical Diabetic Retinopathy and Diabetic Macular Oedema Severity Scale and the UK’s National Screening Committee guidelines.

**Results:**

External evaluation on large datasets collected from three nations (Kenya, Saudi Arabia and China) was carried out. On a DR referable level, sensitivity did not vary significantly between different DR grading schemes (91.2–94.2.0%) and there were excellent specificity values above 93% in all image sets. More importantly, no cases of severe non-proliferative DR, proliferative DR or DMO were missed.

**Conclusions:**

We demonstrate the potential of an AI feature-based DR grading system that is not constrained to any specific grading scheme.

## Introduction

Diabetic retinopathy (DR) is a progressive condition with microvascular complications, which is one of the leading causes of blindness in the working-age population [1]. The classification of disease severity is critical in order to trigger appropriate patient referral to an ophthalmologist. Several classification systems have been developed and adopted in different countries. Two such systems are the ICDRS [2] (The International Clinical Diabetic Retinopathy and Diabetic Macular Oedema Severity Scale) and the NSC standard [3] (National Screening Committee in the UK).

Early diagnosis and treatment through regular screening can slow down disease progression and prevent severe vision loss in diabetic patients. In DR screening programmes, a very large number of digital retinal images need to be examined by human experts. However, significant growth in the number of ophthalmologists or trained human graders is needed in order to meet the demands of an ever-increasing global diabetic population. For example, recent figures indicate that there is a ratio of one ophthalmologist to 100,000 population in India [4] and 1:43,000 in Saudi Arabia [5]; both of which are significantly below the level required to support an effective screening programme.

Automated fundus image analysis offers a potentially efficient reduction in the human workload and could make it economically feasible to scale up screening programmes to the required level globally.

The majority of DR signs that have thus far been characterised (e.g. Microaneurysms (MA), haemorrhages, exudates and intraretinal microvascular abnormalities (IRMA)) can be readily observed in colour fundus photographs. Many computer vision and machine learning algorithms have been applied to automated DR recognition and severity classification [6–11]; typically implemented as Convolutional Neural Networks (CNN) [12]. Deep learning based retinal analysis tools have proved to be reliable in earlier studies [10, 11]. However, end users require provision of more evidence in support of the software’s black-box classification of assigned grade or predictions of progression based on concrete examination findings.

Research on automated DR grading has spanned over 30 years, however the approaches applied to detailed lesion and feature detection remain very limited [6–11]. A key obstacle in the development of feature-based detection is access to a sufficient diversity of well-annotated fundus photographs within publicly available data sources. Our approach has been to work together as a team of computer scientists and clinicians across several different countries, who regularly see patients with diabetic retinopathy, in order to generate well-annotated datasets from a diverse population (both in terms of race and DR severity). Accurate feature-based annotation of our datasets has enabled us to train the system to recognise the underlying clinical features that are diagnostic of DR and its progression as opposed to image-based biomarkers correlated with, but not necessarily caused by, diabetic retinopathy. We have previously shown that this approach facilitates generalisability across diverse global populations [13]. In the current study we demonstrate that the trained system is independent of any one specific grading scheme: Such independence from a specific grading scheme is advantageous as it enables adaptability to the various schemes used by different health care systems around the world. In this paper we assess the performance of the system when applied to both the ICDRS [2] and NSC [3] classifications.

## Methods

Institutional board review at the University of Surrey and participating centres was undertaken and returned a favourable opinion. In addition, research ethics committee review returned a favourable opinion, allowing the study to proceed. Informed consent was obtained from all subjects.

### Predicting severity of retinopathy

In order to allow transparent prediction of the severity grading of an image, we have attempted two approaches:

Whole Image-based: The algorithm is trained to recognise the grade of retinopathy at the global level of whole images using an annotated grading ground truth for each image in the training set. Whilst requiring less detailed annotation of images for training than a feature-based approach, a key weakness is that application to each new grading scheme requires training sets with labels specifically from each scheme.

Feature based: In contrast to the whole image-based approach, the algorithm is trained to recognise specific pathological features. The set of detected features can then be mapped to a corresponding grading level according to a specific classification scheme, and the choice of classification scheme can be changed without any need for retraining: The key advantage is that it provides a flexible grading output based on any given scheme. It also theoretically enables exactly the same feature-detectors to contribute to the diagnosis of other diseases that share features with DR, although the ability to do this was only assessed on an anecdotal basis in the present study. The disadvantage is that it requires detailed fine scale manual annotation of the pathological lesions in order to train the system; we believe this is a key factor that has prevented this approach from being used elsewhere (to our knowledge).

### Measuring progression

Automated DR progression analysis compares two retinal images collected over time and reports on the changes between them. The majority of published work thus far has been limited to a classification of ‘pixel change’ or ‘no pixel change’ between the images [14–16]. Such methods are highly reliant on robust registration across baseline and follow-up images and do not provide a strong indication of pathological evolution. Although some work has been carried out on detecting lesion changes over time using traditional machine learning methods and statistical approaches [17–19], most focus has been on microaneurysms. Only very few studies included other DR features such as exudates, haemorrhages [20] or more advanced pathology. We demonstrate a systematic approach for feature detection from digital colour fundus images, which covers a sufficient diversity of DR features to define severity levels for two different grading classification schemes.

### Training dataset

In this study, we included a training dataset from our own collection along with a public dataset. Our own collection contains 2251 images with detailed annotations of anatomical structures and regions of the pathologies/features present that appear at various levels of DR severity. The public database [21] was obtained using the same 50-degree field-of-view fundus camera with varying imaging settings. The database contained 89 digital retinal images with human expert annotated ground truth for microaneurysms, haemorrhages and exudates. In total, this provided us with 2340 whole images generating over 60,000 sub-image training samples with balanced number of samples across each grade of DR severity. These do not overlap with the testing data used in the validation stage discussed later.

### DAPHNE automated lesion detection

Daphne is an automated system for retinal image analysis, which contains a number of algorithms with a variety of functions. Those related to this particular study are listed below:

1. Image quality assessment, to assess whether or not each individual image was gradable;
2. Anatomical structure segmentation;
3. Lesion detection (measuring presence/absence/number of MAs, haemorrhages, exudates, cotton wool spots (CWS), venous beading/reduplication/loops, IRMA, Neovascularisation at the disk (NVD) neovascularisation elsewhere (NVE), pre-retinal haemorrhage, fibrosis, scaring);
4. Progression analysis and severity rating/grading of the DR and Diabetic Macular Oedema (DMO).

In the first component, a single 10-layer CNN network (inspired by the Oxford Visual Geometry Group [22] and Alexnet [12] network architectures) is applied to assess image quality. This part of the algorithm was trained on about 20,000 unique sample images labelled as readable and unreadable, manually annotated by one or more human experts. Data were first pre-processed to subtract local average colour to reduce differences in lighting [23]. These data were then augmented to increase spatial, rotational and scale variance. To speed up the learning process, batch normalisation and pre-initialisation were applied. The result of the quality assessment is to determine if the image is gradable for further analysis and, if not, it then measures the probability of having any pathological conditions such as cataracts that may affect the quality of the image. If this probability is high, the image is still passed to the next stage. However, images that are assessed as ungradable and with no additional meaningful information, are filtered out as of ‘poor quality’.

In the second component, a set of U-net detectors [24] was trained to detect retinal anatomical structures such as the optic disc, fovea and retinal vessels. The activation function after each convolutional layer was the Rectifier Linear Unit, and a dropout of 0.2 was used between two consecutive convolutional layers. The training was performed on sub-image patches randomly sampled from the whole image, centred on or off the anatomical structures. Further data augmentation was performed to increase spatial, rotational and scale variance. The U-net outputs the locations of anatomical structures in the fundus image.

**Fig. 1:**
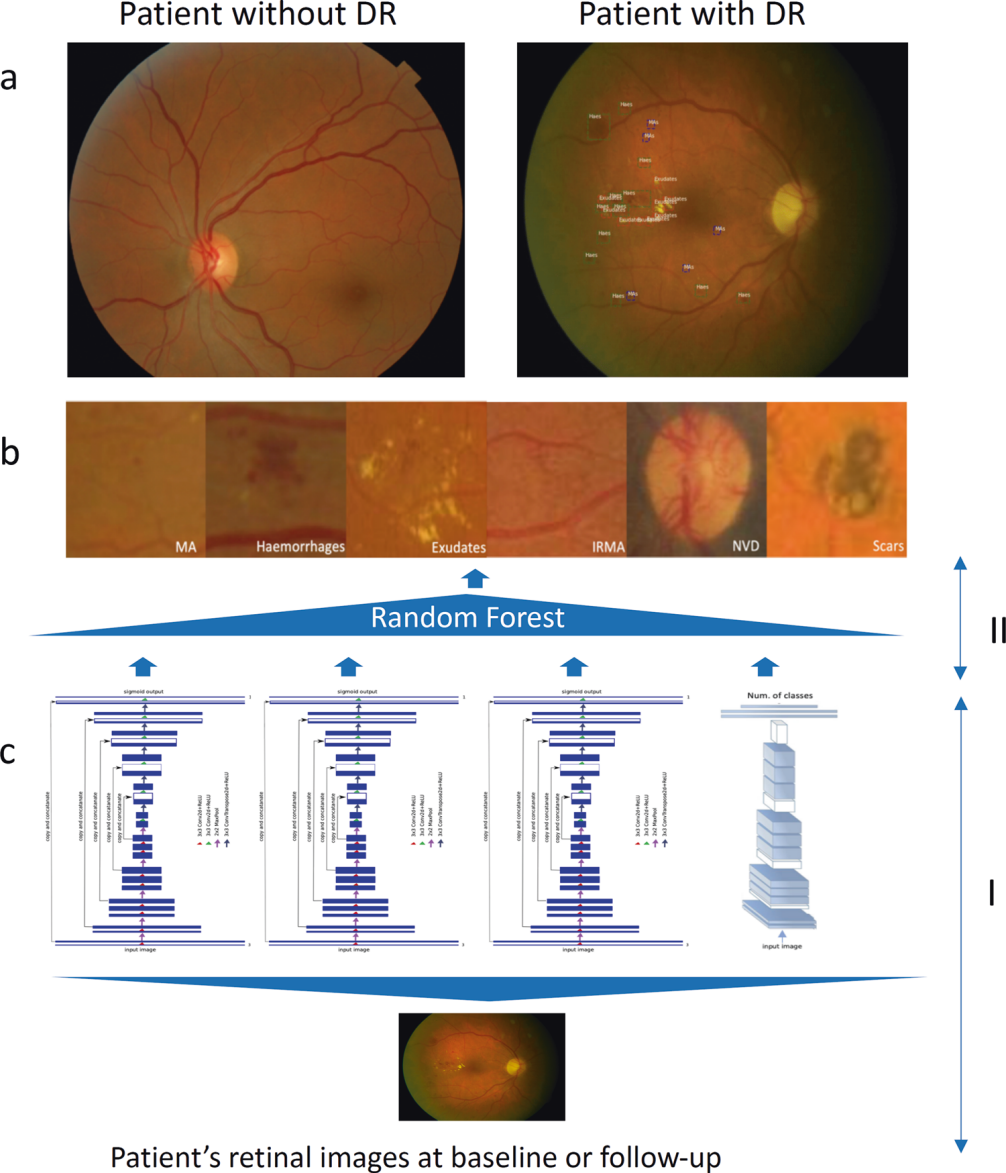
An overview of retinal lesion detectors for grading DR and DMO severity. **a** Sample of fundus images of a patient without DR (left) and a patient with signs of DR (right, MAs, haemorrhage and exudates, are highlighted). The image was graded as moderate in ICDRS or R1 in NSC by the automated system based on these detected features. **b** Samples of annotated lesions by human experts. **c** the two-phase of DR and DMO severity grading: In phase I, the combination of the U-net and CNN-based detectors output whether a certain region is an actual lesion. In phase II, random forest outputs the probabilities of DR and DMO grading.

After extracting the anatomical structures, a combination of U-net and CNN-based lesion detectors was trained to detect the pathological features including MAs, haemorrhages, exudates, CWS, venous beading, venous duplication, venous loop, IRMA, NVD, NVE, pre-retinal haemorrhage, fibrosis and scaring (samples of detected lesions can be seen in Fig. 1b). The U-net and CNN-based detectors output the likelihood that a certain region is an actual lesion. Once anatomical structures and lesions are detected, a random forest-based algorithm determines DR grading (based on ICDRS or UK NSC) and DMO, according to the features detected, their size and location) [25, 26]. Using this feature-based grading scheme easily enables the algorithm to switch between different grading standards, such as the ICDRS and UK NSC grading scales.

**Fig. 2:**
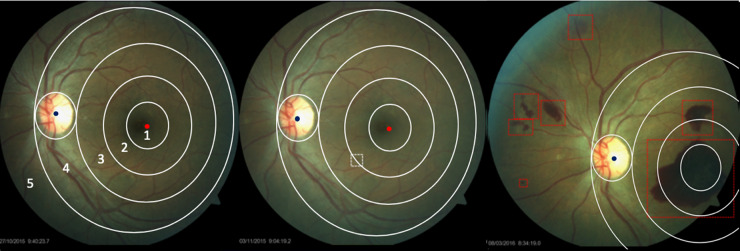
An example of a comparison of morphological changes in DR signs between baseline and follow-up retinal images. Left: A healthy retinal image. It was divided into 5 different regions based on the location of the fovea and the optic disc. Region 1, 2, 3, 4, and 5 are respectively: 1-disc diameter from fovea; between 1 and 1.5-disc diametesr from fovea; between 1.5 and 2-disc diameters from fovea; between 2 and 3-disc diameters from fovea; any other regions. Middle: one month later, one haemorrhage is detected in the region labelled by the white box 2. Right: 5 months later, many more patches of haemorrhages are detected in regions 1, 2, 3 and 5.

When assessing progression, the anatomical structures are first extracted to register baseline and follow-up images for the same eye. These images are partitioned into five regions based on the location of fovea and the optic disc (see Fig. 2). The algorithm then computes any morphological changes in the DR signs for the same eye as per the following measures:

- Any new lesion;
- Any disappearing lesion;
- Any change of existing lesions (smaller or bigger comparing with the baseline images).

When comparing changes between retinal images of the same eye, previous registration methods [14–20] heavily rely on vascular features, which are considered to be reliable structures in the retina. However, some pathological features such as venous beading, neovascularisation and pre-retinal haemorrhage can alter or cover these vascular features over time, giving rise to ambiguity. Including the positions of the optic disc and fovea in the registration process can minimise the misalignment when vessels, as key landmarks, become weaker or less reliable.

## Results

### Statistical analysis of performance

In this study, our system was used to detect DR lesions and then grade the images on the basis of the features detected, including categorisation of referable DR based on a given grading scheme. We also evaluated DAPHNE’s performance on three validation sets of images. The evaluation included measuring accuracy, sensitivity, specificity and the 95% confidence intervals. We also calculated the DR grading agreement between the automated system and human experts using quadratic weighted kappa. Moreover, sensitivity, specificity and AUC values for detecting DMO and progression changes were calculated. All analyses were measured through the StatsModels version 0.8.0 and SciPy version 1.0.0 Python data analysis packages.

### Automated grading of images using features

Our hypothesis is that by training our system to identify features, we are not constrained to any one specific grading scale. To test this, we evaluated DAPHNE’s ability to match results from human graders using the NSC or ICDRS grading scales.

The evaluation image-sets contained 49,726 images from three nations: Kenya, Saudi Arabia, and China. None of these images were used for training, and the prediction algorithm had no prior knowledge on their grading level either on the NSC or ICDRS schemes. For evaluation, we divided the total set into two. The first set from Kenya contained 28680 images that had been annotated using the ICDRS scheme by trained graders. The second set contained 10,026 images from Saudi Arabia and 15,000 images from China, both annotated in NSC. The prevalence of DR severities is shown in Table 1. The evaluation measured both the ability of DAPHNE to match assessments using different grading schemes and the generalisability of the model to diverse populations.

**Table 1:** The prevalence of DR severities in three datasets.

Graded in NSC	R0	R1	R2	R3	
China	9986	3279	1240	495	
Saudi Arabia	7451	1854	582	139	
Graded in ICDRS	0	1	2	3	4
Kenya	13304	10967	3935	381	93

We used a quadratic weighted kappa score, sensitivity and specificity to calculate the agreement between the lesion detectors with human annotations. Here are the definitions of some key measures:

True positive (TP): Contains at least one correct lesion within the severity level against ground truth (GT) which indicates the grading level.

True negative (TN): Nothing is detected when the image is normal; or non-referable features are detected if the image is non-refer. For example, if an image is mild grade in ICDRS, and only MAs are detected, then it is TN.

False positive (FP): Detects lesions that belong to a higher severity level than GT level.

False negative (FN): Only find lesions belonging to lower severity level than GT.

### Grading assessment accordingly to ICDRS

The Kenya dataset consisted of 28,680 images, two images for each eye, and some of the eyes had two baseline and follow-up images over a five-year period. 94% of the images were defined as gradable by the automated system.

The automated system obtained a quadratic weighted kappa score of 0.85 indicating excellent agreement. The performance summaries for severity level detection according to the ICDRS scale (DR vs Non-DR, Referral vs Non- Referral and PDR vs Non-PDR) are as follows: For the DR vs Non-DR levels, the sensitivity of our system was 91.19% (95% CI: 90.2–92.2%) and specificity was 94.6% (95% CI: 94.2–94.8%). For the referable DR level, the sensitivity was 92.03% (95% CI: 91.2–92.9%) and specificity was 93.0% (95% CI: 92.7–93.4%). For the PDR level, the sensitivity was 100% (meaning no cases of PDR cases were missed) and specificity was 86.7% (95% CI: 86.2–87.1%).

In this study, there were 1264 patients with baseline and follow-up retinal images. For the DR progression changes between baseline and follow-up images, when the severity of baseline retinal images was higher than moderate NPDR, the sensitivity of our system was 100%. Moreover, our system did not miss any cases when PDR developed. We observed that the system detected some other non-DR lesions and individual artefacts as DR lesions, and therefore needs further training in order to minimise FP and differentiate other lesions that are similar to DR.

### Grading assessment according to UK NSC

A further set of data consisted of 10,026 Saudi Arabian and 15,000 Chinese fundus images, two for each eye, one of which was optic disc centred and the other macula-centred. The Saudi cohort had two baseline and multiple follow-up retinal images for each eye. 92% of the images were classified as gradable by the software system. Figure 2 shows the results of the algorithm for lesion detection on a case with images taken over time to monitor progression.

The automated system obtained a quadratic weighted kappa score of 0.83. Table 2A summarises the performance for the detection of different severity levels according to the UK NSC standard (DR vs Non-DR, Referral vs Non- Referral, PDR vs Non-PDR and DMO). For the DR vs Non-DR levels, the sensitivity of our system was 92.59% (95% CI: 91.2–93.3%) and specificity was 93.0% (95% CI: 92.7–93.84). For the referable DR level, the sensitivity of our system was 92.9% (95% CI: 91.4–94.2%) and the specificity was 94.4% (95% CI: 94.1–97.8%). For the PDR level, the sensitivity of our system was 100% (which means no cases of PDR cases were missed) and the specificity was 87.1% (95% CI: 86.7–87.6%). For the DMO, the sensitivity of our system was 100% (which means no cases of DMO were missed) and specificity was 84.2% (95% CI: 83.8–84.7%).

**Table 2:** Sensitivity, specificity and corresponding 95% CIs for different disease levels (A) for datasets graded with the NSC scheme, (B) for the Kaggle dataset (35,124 images).

A:				
Disease Level	DR vs Non-DR	Referral vs Non- Referral	PDR vs Non-PDR	DMO
Sensitivity	92.59% (91.86–93.28%)	92.83% (91.38–94.16%)	100%	100%
Specificity	93.02% (92.67–93.36%)	94.42% (94.10–94.72%)	87.12% (86.69–87.55%)	84.23% (83.77–84.69%)

B:			
Disease Level	DR vs Non-DR	Referral vs Non- Referral	PDR vs Non-PDR
Sensitivity	91.23% (87.15–94.15%)	92.21% (86.36–95.0%)	98.65% (91.5–99.6%)
Specificity	92.91% (91.2–95.1%)	96.9% (95–97.85%)	85.78% (83.9–88.7%)

The 10,026 baseline and follow-up retinal images were from 501 Saudi cohort subjects whose DR progression was being monitored. Of those baseline images with severity R3 or M1, the sensitivity of our system was 100%. Moreover, the automated system did not miss any cases of PDR or M1 DR.

### Further testing on the Kaggle dataset with evidence-based DR grading

**Fig. 3:**
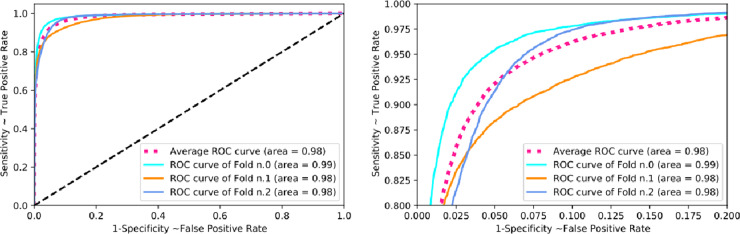
ROC for referable DR in Kaggle Dataset. ROC (receiver operating characteristic) curve for referable diabetic retinopathy in the Kaggle Dataset with different prevalence of referable cases (5% (*n*. 0), 15% (*n*. 1) and 30.5% (*n*. 2)), using feature-based grading. Right is zoom-in version of the left.

We further evaluated our feature-based grading approach on the Kaggle dataset [27]. This set consisted of 35,124 images as its original training set, graded using ICDRS with one image for each eye. We used these labelled samples to test our feature-based approach. 77% of images were classified as gradable by the automated system. Three experiments were carried out: The original referable prevalence in Kaggle dataset was 30.5%. We further randomly selected referable images to allow respectively 5% and 15% referable prevalence and compared the performance on these three prevalence scenarios as illustrated in Fig. 3 and Table 2b.

Regarding the detection of referable DR (based on the ICDRS), two operating points were selected, one for high sensitivity and another for high specificity. At high sensitivity operating points, the sensitivity of our system was 94.6% (95% CI: 93.8–94.9%) and specificity was 75.5% (95% CI: 75.1–76.2%), a negative predictive value of 98.3% (95% CI: 98.2–98.4%), and positive predictive value of 48.6% (95% CI: 48.2–49.1%). At high specificity operating points, the sensitivity of our system was 81.1% (95% CI: 80.5–81.8%) and specificity was 92.8% (95% CI: 92.5–92.9%), a negative predictive value of 95.4% (95% CI: 95.2–95.5%), and positive predictive value of 72.3% (95% CI: 71.6–72.9%). The AUC for the detection of referable DR was 0.98 (95% CI: 0.969–0.993%).

Our system obtained a quadratic weighted kappa score of 0.857 for grading according to the ICDR scale, which is slightly lower than the winner of the DR competition, but higher than other published methods. However, unlike the published end-to-end paradigms that only make predictions based on the whole image, the grading approach in our platform is generated from the detected features in a similar fashion to the human grading process.

### Detection of co-pathology

The ability of DAPHNE to detect non-DR co-pathology was assessed for a small sample of selected cases with pathology that was clearly visible on the fundus photograph. Of 1196 cases with age related macular degeneration, 945 were detected. Of seven cases with papilledema all were detected. Of two cases with lattice degeneration, both were detected. Of two cases with retinal detachment, both were detected. Of 13 cases with peri-papillary atrophy, 12 were detected. Of two cases with macular holes, both were detected. Of 14 cases of epiretinal membrane, 13 were detected. Of two cases of CRVO, both were detected. A formal assessment of sensitivity and specificity was not performed given the small numbers involved in this pilot-study arm of the project.

## Discussion

In this study we demonstrate a systematic, feature-based methodology for diabetic retinopathy and macular oedema detection and evaluate its performance according to two grading standards: ICDRS and UK NSC. Our regional detection deep learning model achieved good results on image sets from a large dataset containing images from different camera types and with varying settings. In all datasets the overall agreement between DAPHNE and human grading was above 85%. Hence, we demonstrate that our AI software has the ability to perform DR grading and progression monitoring with high accuracy. More importantly the software did not miss any sight-threatening cases in any of the studies.

Whilst our earlier work demonstrated the generalisability of DAPHNE over different global populations, this work demonstrates that the system will also generalise to different grading schemes without any need for retraining. In addition, early work indicates that it can sometimes identify other common eye conditions (such as age-related macular degeneration, epiretinal membrane, retinal detachment and optic disc abnormalities) where signs are visible on colour fundus photography, however the sensitivity and specificity of these findings has not been formally assessed in the present paper and this will be the subject of future work.

Some false positives were, however, present due to the system’s overestimation of the severity of the DR (a higher grade of DR than was actually present). Other types of pathologies and individual artefacts were detected as DR-related and produced false positive outcomes contributing to the overestimate. Further refinement of the training as well as a systematic diagnostic pathway taking into account all potential co-pathology should be considered.

The key capability that distinguishes our system from other state-of-the-art methods, is that our system can provide the clinically relevant details that support grading results and therefore, in contrast to the ‘black box’ approach, users can check how the conclusion was reached in each case. In some cases where other non-DR pathologies exist, our system was able to locate the abnormal regions just as human DR graders may do and developing this capability will be the subject of future work in our lab.

In conclusion, this study shows the potential of an AI software for feature-based DR grading and progression assessment that is not tied to any specific grading scheme. If used as a pre-screening filter, a 70% reduction of the number of patients needing to be graded by humans could be achieved assuming that only positive returns are seen again by a human. Further development on other common eye conditions will continue to facilitate the software’s usefulness as a tool in assisting clinical services.

## Summary

### What was known before

- End to end AI grading of diabetic retinopathy can reliably classify disease by severity.

### What this study adds

- By designing an AI grading system based on feature detection rather than end to end ‘whole image’ analysis, we have developed software that is transferable between different grading systems and whose decisions can be more easily compared with those of clinicians.
